# Supplementary material for: Few-shot learning for inference in medical imaging with subspace feature representations
Source: PLoS One. 2024 Nov 6;19(11):e0309368. doi: 10.1371/journal.pone.0309368 (PMC11540231; doi:10.1371/journal.pone.0309368)
Supplement: S1 File — (PDF) [file pone.0309368.s001.pdf]

Supporting Information

S1 Appendix. Discriminant Directions

S1.1 Derivation of the Second Discriminant Direction

This Appendix derives an important step in the derivation of the second discriminant direction $\boldsymbol{d}_2$, which is not shown in [1, 2].

Maximizing the objective function in Eq. (9) in Section 2.4.2 with respect to $\boldsymbol{d}_2$ can be addressed by solving

$$\frac{2\tilde{\boldsymbol{S}}_{\mathrm{B}}\boldsymbol{d}_2}{\boldsymbol{d}_2^\top \tilde{\boldsymbol{S}}_{\mathrm{W}}\boldsymbol{d}_2} - \frac{2\boldsymbol{d}_2^\top \tilde{\boldsymbol{S}}_{\mathrm{B}}\boldsymbol{d}_2\tilde{\boldsymbol{S}}_{\mathrm{W}}\boldsymbol{d}_2}{(\boldsymbol{d}_2^\top \tilde{\boldsymbol{S}}_{\mathrm{W}}\boldsymbol{d}_2)^2} - \lambda\boldsymbol{d}_1 = 0. \tag{17}$$

Note that $\tilde{\boldsymbol{S}}_{\mathrm{B}} = \boldsymbol{s}_{\mathrm{b}}\boldsymbol{s}_{\mathrm{b}}^\top$. Substituting it into Eq. (17) yields

$$\frac{2\boldsymbol{s}_{\mathrm{b}}\boldsymbol{s}_{\mathrm{b}}^\top\boldsymbol{d}_2}{\boldsymbol{d}_2^\top \tilde{\boldsymbol{S}}_{\mathrm{W}}\boldsymbol{d}_2} - \frac{2\boldsymbol{d}_2^\top \boldsymbol{s}_{\mathrm{b}}\boldsymbol{s}_{\mathrm{b}}^\top\boldsymbol{d}_2\tilde{\boldsymbol{S}}_{\mathrm{W}}\boldsymbol{d}_2}{(\boldsymbol{d}_2^\top \tilde{\boldsymbol{S}}_{\mathrm{W}}\boldsymbol{d}_2)^2} - \lambda\boldsymbol{d}_1 = 0. \tag{18}$$

Let $\kappa = \boldsymbol{s}_{\mathrm{b}}^\top\boldsymbol{d}_2/\boldsymbol{d}_2^\top\tilde{\boldsymbol{S}}_{\mathrm{W}}\boldsymbol{d}_2$, which is a scalar. Eq. (18) can be rewritten as

$$2\kappa\boldsymbol{s}_{\mathrm{b}} - 2\kappa^2\tilde{\boldsymbol{S}}_{\mathrm{W}}\boldsymbol{d}_2 - \lambda\boldsymbol{d}_1 = 0. \tag{19}$$

Then we have

$$\boldsymbol{d}_2 = \frac{1}{\kappa}\tilde{\boldsymbol{S}}_{\mathrm{W}}^{-1}\left(\boldsymbol{s}_{\mathrm{b}} - \frac{\lambda}{2\kappa}\boldsymbol{d}_1\right). \tag{20}$$

Since $\boldsymbol{d}_1 = \tilde{\boldsymbol{S}}_{\mathrm{W}}^{-1}\boldsymbol{s}_{\mathrm{b}}$, we get

$$\boldsymbol{d}_2 = \frac{1}{\kappa}\left(\tilde{\boldsymbol{S}}_{\mathrm{W}}^{-1} - \frac{\lambda}{2\kappa}\left(\tilde{\boldsymbol{S}}_{\mathrm{W}}^{-1}\right)^2\right)\boldsymbol{s}_{\mathrm{b}}. \tag{21}$$

Let $S_{11} = \boldsymbol{d}_1^\top\tilde{\boldsymbol{S}}_{\mathrm{W}}^{-1}\boldsymbol{d}_1$. Since $\boldsymbol{d}_1^\top\boldsymbol{d}_2 = 0$, we have

$$\boldsymbol{d}_1^\top\boldsymbol{d}_2 = \frac{1}{\kappa}\boldsymbol{d}_1^\top\tilde{\boldsymbol{S}}_{\mathrm{W}}^{-1}\boldsymbol{s}_{\mathrm{b}} - \frac{\lambda}{2\kappa^2}\boldsymbol{d}_1^\top\tilde{\boldsymbol{S}}_{\mathrm{W}}^{-1}\boldsymbol{d}_1 = 0, \tag{22}$$

which gives

$$\frac{1}{\kappa}\boldsymbol{d}_1^\top\boldsymbol{d}_1 - \frac{\lambda}{2\kappa^2}S_{11} = 0. \tag{23}$$

Therefore,

$$\frac{\lambda}{2\kappa} = \frac{\boldsymbol{d}_1^\top\boldsymbol{d}_1}{S_{11}}. \tag{24}$$

Since

$$\begin{aligned} S_{11} &= \boldsymbol{d}_1^\top\tilde{\boldsymbol{S}}_{\mathrm{W}}^{-1}\boldsymbol{d}_1 \\ &= \boldsymbol{s}_{\mathrm{b}}^\top\tilde{\boldsymbol{S}}_{\mathrm{W}}^{-1}\tilde{\boldsymbol{S}}_{\mathrm{W}}^{-1}\tilde{\boldsymbol{S}}_{\mathrm{W}}^{-1}\boldsymbol{s}_{\mathrm{b}}, \\ &= \boldsymbol{s}_{\mathrm{b}}^\top\left(\tilde{\boldsymbol{S}}_{\mathrm{W}}^{-1}\right)^3\boldsymbol{s}_{\mathrm{b}}, \end{aligned} \tag{25}$$

and $\boldsymbol{d}_1^\top\boldsymbol{d}_1 = \boldsymbol{s}_{\mathrm{b}}^\top\left(\tilde{\boldsymbol{S}}_{\mathrm{W}}^{-1}\right)^2\boldsymbol{s}_{\mathrm{b}}$, we have

$$\frac{\lambda}{2\kappa} = \frac{\boldsymbol{s}_{\mathrm{b}}^\top\left(\tilde{\boldsymbol{S}}_{\mathrm{W}}^{-1}\right)^2\boldsymbol{s}_{\mathrm{b}}}{\boldsymbol{s}_{\mathrm{b}}^\top\left(\tilde{\boldsymbol{S}}_{\mathrm{W}}^{-1}\right)^3\boldsymbol{s}_{\mathrm{b}}}. \tag{26}$$

Substituting it into Eq. (21), we have

$$\boldsymbol{d}_2 = \frac{1}{\kappa}\left(\tilde{\boldsymbol{S}}_{\mathrm{W}}^{-1} - \frac{\boldsymbol{s}_{\mathrm{b}}^\top(\tilde{\boldsymbol{S}}_{\mathrm{W}}^{-1})^2\boldsymbol{s}_{\mathbf{b}}}{\boldsymbol{s}_{\mathrm{b}}^\top(\tilde{\boldsymbol{S}}_{\mathrm{W}}^{-1})^3\boldsymbol{s}_{\mathrm{b}}}(\tilde{\boldsymbol{S}}_{\mathrm{W}}^{-1})^2\right)\boldsymbol{s}_{\mathrm{b}}. \tag{27}$$

Normalise $\boldsymbol{d}_2$ above, which completes the derivation.

S2 Appendix. Supervised NMF

The supervised NMF suggested by [3], introducing a logistic regression model into the cost function of NMF in Eq. (15). Let $\boldsymbol{Z} := \left[1 \mid \boldsymbol{Y}\boldsymbol{X}^\top\right] = (\boldsymbol{z}_1, \boldsymbol{z}_2, \cdots, \boldsymbol{z}_N)^\top \in \mathbb{R}^{N\times(p+1)}$. Note that $\boldsymbol{z}_i \in \mathbb{R}^{p+1}, 1 \leq i \leq N$. Considering the binary classification problem, let $\boldsymbol{u} \in \{0,1\}^N$ be the vector representing the labels (i.e., 0 or 1) of the given N samples. Let $\boldsymbol{\beta} \in \mathbb{R}^{p+1}$. The total loss function of the supervised NMF is defined as

$$\min_{\boldsymbol{K},\boldsymbol{X}\geq 0,\boldsymbol{\beta}} \frac{1}{2}\|\boldsymbol{Y} - \boldsymbol{K}\boldsymbol{X}\|_F^2 + \frac{\tilde{\lambda}}{N}\Big(\sum_{i=1}^{N}\log\left(1+\exp(\boldsymbol{z}_i^\top\boldsymbol{\beta})\right) - \boldsymbol{u}^\top\boldsymbol{Z}\boldsymbol{\beta}\Big), \tag{28}$$

where $\tilde{\lambda} \geq 0$ is the regularization parameter. Problem (28) can be minimised corresponding to $\boldsymbol{K}, \boldsymbol{X}$ and $\boldsymbol{\beta}$ alternatively. Minimisation with respect to $\boldsymbol{K}$ has the same update rules with (16). Because of the additional included logistic regression term in the loss function, we follow the stochastic gradient descent ADADELTA used in [3] to update $\boldsymbol{X}$ and $\boldsymbol{\beta}$. Method ADADELTA leverages the knowledge from previously computed gradients to determine the required gradients $\Delta\boldsymbol{X}$ and $\Delta\boldsymbol{\beta}$ in the next step. To guarantee non-negativity of $\boldsymbol{X}$, one projection step, denoted by $\mathrm{proj}(\boldsymbol{X})$, is added to replace all entries of the $\boldsymbol{X}$ less than 0 with a small positive number. The whole update rules for supervised NMF is

$$\boldsymbol{K} \leftarrow \boldsymbol{K} \circ \frac{\boldsymbol{Y}\boldsymbol{X}^\top}{\boldsymbol{K}\boldsymbol{X}\boldsymbol{X}^\top}, \quad \boldsymbol{X} \leftarrow \mathrm{proj}(\boldsymbol{X}) + \boldsymbol{\Delta X}, \quad \boldsymbol{\beta} \leftarrow \boldsymbol{\beta} + \boldsymbol{\Delta\beta}. \tag{29}$$

For more details please see [3].

S3 Appendix. Experiment Supplement

S3.1 Comparison with Manifold Learning

A manifold in mathematics is a topological space that resembles Euclidean space. One of the most commonly used manifold learning methods is the nonlinear dimensionality reduction technique Isomap [4]. Isomap is used for computing a quasi-isometric and low-dimensional embedding of a set of high-dimensional data points. Comparison between NMF, SVD and Isomap subspaces in terms of classification accuracy on the 14 datasets as the subspace dimensions ranging from 1 to 70 is given in S3 Fig 8. Ten random partitions of the training-test set on each of the 14 datasets are conducted. It shows that in many cases NMF subspace exhibits slightly better performance in different dimensions than the SVD subspace. Moreover, both SVD and NMF outperform the Isomap subspace by a large margin. The comparison between DA, SVD and Isomap subspaces in terms of classification accuracy on all the datasets is given in S3 Fig 9. It shows that Isomap and SVD produce similar results in low dimensions, while DA is able to achieve better results than both Isomap and SVD by a large margin. On the whole, the above results again suggest the viable alternatives of using NMF and DA to the SVD-based low dimensional subspaces.

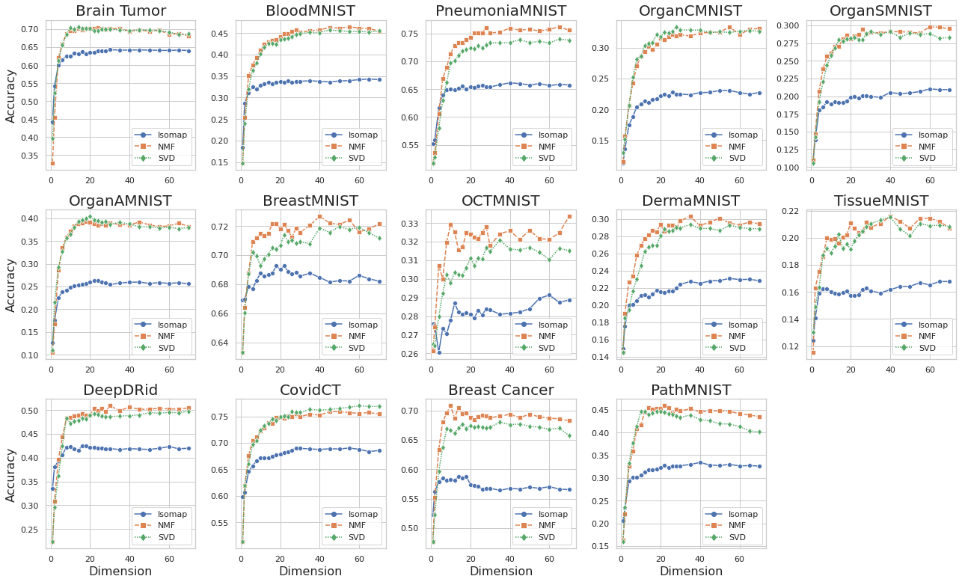

Fig 8. Classification accuracy comparison between the SVD, Isomap and NMF subspaces on 14 datasets with subspace dimensions ranging from 1 to 70. Ten random partitions of the training-test set on each of the 14 datasets are conducted. It shows that in many cases NMF subspace exhibits slightly better performance in different dimensions than the SVD subspace. Moreover, both SVD and NMF outperform the Isomap subspace by a large margin.

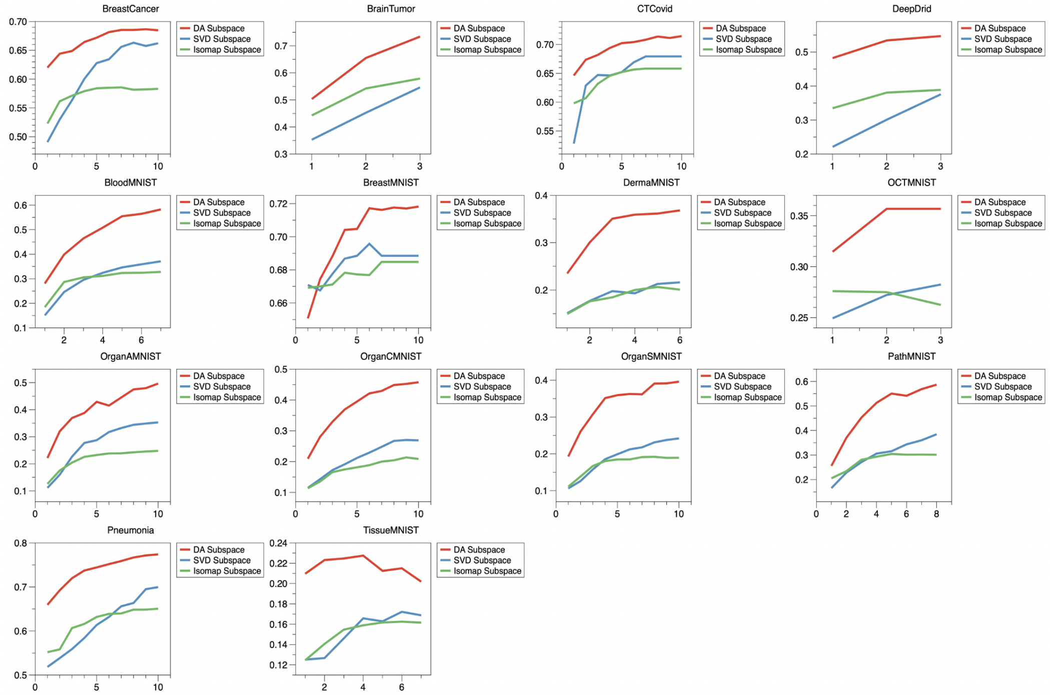

Fig 9. Classification accuracy comparison between the SVD, Isomap and DA subspaces on the 14 datasets. It shows that DA outperforms both SVD and Isomap by a large margin.

S3.2 Boruta Results

S3 Table 5 gives the classification results based on Boruta feature selection and the average number of selected features across ten runs for each of the 14 medical datasets, from which we see that the Boruta approach is highly unstable (i.e., yielding large deviation).

Table 5. Few-shot learning classification accuracy by Boruta feature selection on the 14 medical datasets.

Datasets	Accuracy	Selected Features	Classes
`BreastCancer` [5]	65.16±5.21	32	2
`BrainTumor` [6]	69.77±3.58	284	4
`CovidCT` [7]	72.63±2.82	58	2
`DeepDRiD` [8]	49.70±4.84	106	5
`BloodMNIST` [9]	42.08±2.83	183	8
`BreastMNIST` [10]	69.50±4.39	13	2
`DermaMNIST` [11]	26.66±4.06	41	7
`OCTMNIST` [12]	30.28±3.01	19	4
`OrganAMNIST` [13]	37.36±1.99	170	11
`OrganCMNIST` [13]	30.62±2.85	125	11
`OrganSMNIST` [13]	26.24±2.21	100	11
`PathMNIST` [14]	38.58±2.58	197	9
`PneumoniaMNIST` [12]	75.78±3.79	71	2
`TissueMNIST` [15]	19.23±3.61	26	8

S3.3 Classification Results via SVM

S3 Fig 10 depicts the performance of applying SVM as a classifier in the developed few-shot learning framework using the same setup as KNN on the 14 medical datasets. In particular, we implemented both the rbf kernel and the linear kernel for SVM. The C and gamma parameters were also fine-tuned for different datasets. The same dimensions as NMF and DA were applied to SVD to ensure fair comparison. We discovered that the rbf kernel worked better in comparisons between SVD and DA in low dimensions, and the linear kernel performed better in comparisons between SVD and NMF in medium dimensions. On the whole, consistent results were obtained by using SVM and KNN as classifiers on the 14 distinct medical datasets, indicating that the developed few-shot learning architecture is robust to the choice of classifiers.

S3.4 T-SNE Visualization

For better visual validation, S3 Fig 11 shows an example of visualising the subspaces utilising the T-SNE visualization technique (built-in function in Python) on the brain tumour dataset. S3 Fig 11(a) and 11(b) are the 2D feature visualization of the original feature space (i.e., features extracted by the pre-trained network) and the DA subspace, respectively. S3 Fig 11(c) and 11(d) show the features that are projected to the 30-dimensional subspace by SVD and NMF, respectively. These plots visually prove that the DA and NMF subspaces are indeed viable alternatives to SVD.

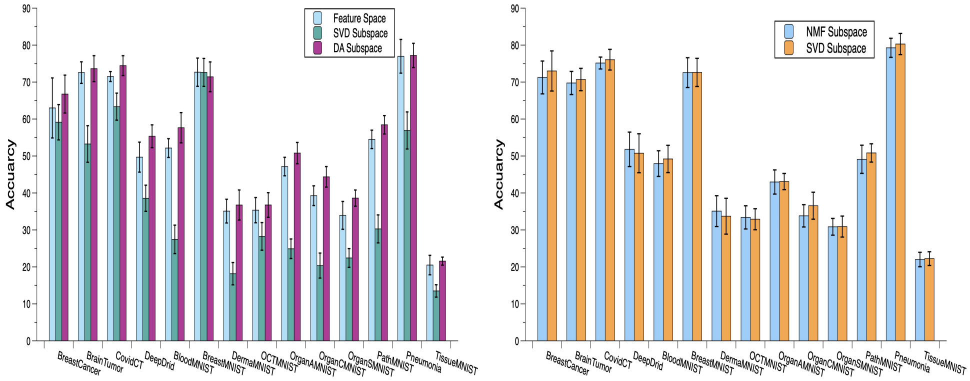

Fig 10. Few-shot learning performance using SVM as classifier on the 14 medical datasets. Left panel: results obtained by using the original feature space, the SVD subspace and the DA subspace. Right panel: results obtained by using the features in 30-dimensional subspace derived by SVD and NMF.

S3.5 Comparison with the Prototypical Network

Below we compare our techniques with the well-known few-shot learning algorithm, the prototypical network [16], on all the 14 medical datasets, see S3 Table 6. The architecture of the prototypical network used in this experiment is the same as the one in [16], which is composed of four convolution blocks and has been trained on the omniglot dataset [17] via SGD with Adam optimiser [18] and obtained 99% accuracy in the 5-shot scenario. Each block in this network is comprised of a 64-filter 3×3 convolution, batch normalisation layer, a ReLU nonlinearity and a 2×2 max-pooling layer. The classification accuracy reported in S3 Table 6 is averaging over 10 randomly generated episodes from the test set. The setting for the test experiment is "C-way 5-shot," where 5 samples are given for each class in the support set and C is the number of classes in each dataset. To validate the final performance, 15 query images per class are provided. Recall that the original feature space represents the features extracted by the network without dimensionality reduction. The dimensions for the NMF/SVD subspaces are chosen as 2, 5, 10, 20, 30, 40 and 50, separately. The dimensions of the DA subspace are chosen as 10 for the binary classification problem and $(C - 1)$ for the multiclass classification problem. The results in S3 Table 6 demonstrate that our method outperforms all the other methods, i.e., the prototypical network and the ones with the original feature space and SVD.

S3.6 Impact of the Dataset Size and Dimensionality

S3 Fig 12 shows the impact of the dataset size on the classification accuracy of NMF and SVD as the subspace dimension changes, where datasets `BloodMNIST` with eight classes and `DeepDRid` with five classes are used. The setting in S3 Fig 12 is the same as the one used in Fig 4. It shows that, in the multiclass classification problem, fewer categories will result in higher accuracy, and SVD suffers from dimension changes more in the datasets with small size. Without enough data, SVD could not extract specific precise features that match the target categories or may extract insignificant features as the dimension increases, resulting in low classification performance (e.g. see S3 Fig 12(b). In contrast, the results of NMF are robust to dimension changes in datasets with different size. This great performance of NMF benefits from its part-based representation maximising the features preserved in the subspace.

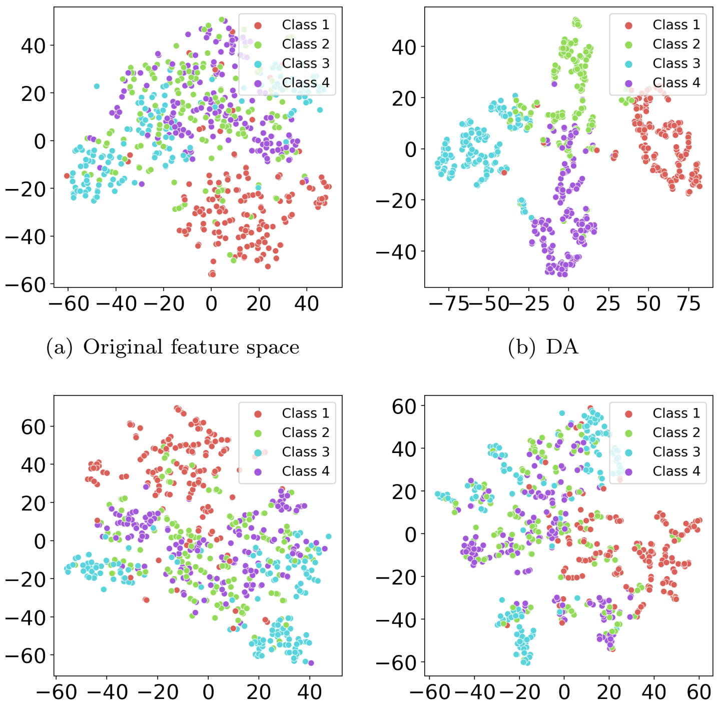

(a) Original feature space (b) DA

(c) SVD (d) NMF

Fig 11. Subspace visualization by T-SNE on the brain tumour dataset. (a)–(d): the results regarding the originally feature space, DA subspace, 30-dimensional subspace derived by SVD and NMF, respectively.

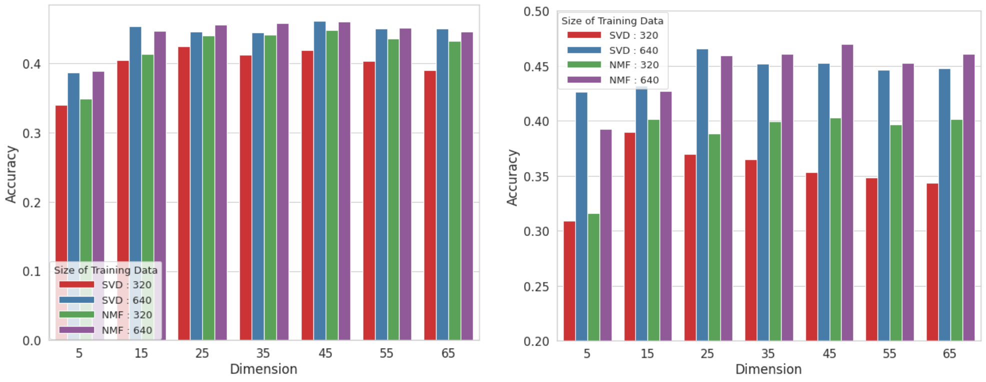

(a) `BloodMNIST` (b) `DeepDRid`

Fig 12. Comparison between NMF and SVD subspaces in terms of classification accuracy corresponding to different dataset size as the subspace dimension changes. Datasets `BloodMNIST` with eight classes and `DeepDRid` with five classes are used in panels (a) and (b), respectively.

Table 6. Classification accuracy comparison between the prototypical network and the few-shot learning with subspace feature representations.

C-way 5-shot Accuracy(%)										
	Methods									
Data	Prototypical	Few-shot Learning with Subspace Feature Representations (**Ours**)								
	Network	Feature Space	Subspaces	2 Dim	5 Dim	10 Dim	20 Dim	30 Dim	40 Dim	50 Dim
CovidCT (2 classes)	53.33±6.99	52.22±5.88	SVD	49.78±10.95	54.56±5.80	52.11±5.32	52.11±5.32	52.11±5.32	52.11±5.32	52.11±5.32
			NMF	53.00±10.05	**56.89±8.07**	56.44±4.89	55.89±6.61	55.78±6.36	56.33±6.09	56.44±6.08
			DA	51.91±3.41 (10 Dim)						
BreastCancer (2 classes)	72.33±8.68	70.22±8.68	SVD	66.89±5.83	70.33±8.61	70.11±9.07	70.11±9.07	70.11±9.07	70.11±9.07	70.11±9.07
			NMF	68.78±7.83	72.44±7.71	72.78±9.69	72.11±7.82	**73.00±8.93**	71.33±9.27	72.44±8.37
			DA	62.69±4.43 (10 Dim)						
PneumoniaMNIST (2 classes)	64.33±8.17	66.56±9.79	SVD	61.56±9.34	66.11±7.32	66.56±8.92	66.56±8.92	66.56±8.92	66.56±8.92	66.56±8.92
			NMF	62.67±9.44	70.33±6.38	72.00±6.46	72.89±6.03	72.56±5.75	73.33±7.42	**73.78±6.78**
			DA	68.45±5.79 (10 Dim)						
BreastMNIST (2 classes)	54.00±9.40	54.11±6.43	SVD	54.00±7.14	54.11±6.67	54.67±7.47	54.67±7.47	54.67±7.47	54.67±7.47	54.67±7.47
			NMF	53.00±7.70	59.11±4.63	60.33±5.17	60.56±3.66	59.00±6.84	60.00±6.19	**62.33±5.45**
			DA	59.98±8.08 (Dim = 10)						
DeepDRid (5 classes)	30.07±7.87	29.47±4.91	SVD	27.58±4.53	28.03±5.59	29.42±4.75	29.28±4.95	29.47±4.98	29.47±4.98	29.47±4.98
			NMF	28.92±4.51	30.03±5.59	31.07±4.48	31.27±4.59	31.02±4.54	**31.47±4.17**	31.37±4.07
			DA	**31.63±7.07** (4 Dim)						
BrainTumor (4 classes)	34.67±4.82	35.42±5.29	SVD	33.12±4.69	34.54±4.84	35.00±5.29	35.42±5.23	35.42±5.23	35.42±5.23	35.42±5.23
			NMF	33.96±5.45	36.04±4.92	36.88±4.73	37.33±5.20	**37.87±5.10**	37.50±5.10	37.46±4.87
			DA	**61.88±5.50** (3 Dim)						
BloodMNIST (8 classes)	47.58±5.24	48.29±2.52	SVD	36.29±2.98	46.42±4.65	47.62±2.49	48.06±3.15	**48.33±2.45**	48.33±2.63	48.33±2.63
			NMF	36.60±3.77	47.33±4.96	46.94±4.42	47.02±3.87	46.23±2.71	45.90±3.65	46.58±3.56
			DA	**54.33±8.08** (7 Dim)						
DermaMNIST (7 classes)	26.57±4.69	25.83±3.57	SVD	21.43±4.61	25.07±3.63	25.19±3.28	25.33±3.67	25.71±3.57	25.76±3.58	25.76±3.58
			NMF	22.71±3.49	27.45±4.02	26.19±3.36	26.55±3.16	27.86±3.40	26.83±2.92	**27.79±2.98**
			DA	**31.14±5.54** (6 Dim)						
OCTMNIST (4 classes)	28.64±3.95	29.79±3.02	SVD	26.08±3.80	28.50±4.31	29.42±2.94	29.83±3.43	29.83±3.43	29.83±3.43	29.83±3.43
			NMF	26.12±3.63	31.17±2.52	**32.17±3.12**	31.75±3.20	32.13±3.49	31.04±3.16	32.12±3.57
			DA	**32.92±6.68** (3 Dim)						
OrganAMNIST (11 classes)	47.94±2.39	52.50±3.03	SVD	34.26±3.28	45.52±3.92	50.03±3.36	52.73±3.29	52.74±3.48	52.89±3.15	52.71±3.22
			NMF	33.70±3.38	45.39±2.67	53.00±3.31	53.88±3.38	**53.88±3.26**	52.97±2.82	53.58±3.99
			DA	**60.94±3.86** (10 Dim)						
OrganCMNIST (11 classes)	48.55±4.11	50.23±3.84	SVD	30.61±3.48	42.12±4.15	48.68±3.71	49.70±4.23	50.18±4.46	50.18±4.02	50.08±4.25
			NMF	29.86±2.77	40.55±3.83	49.55±3.67	51.92±4.72	**52.29±4.40**	51.26±4.55	50.91±3.55
			DA	**60.62±3.10** (10 Dim)						
OrganSMNIST (11 classes)	34.67±4.21	36.95±3.69	SVD	25.74±2.64	33.79±3.32	35.70±4.01	36.52±3.86	**37.18±3.43**	37.05±3.45	36.94±3.58
			NMF	24.67±2.41	33.97±3.04	36.06±3.24	36.45±3.11	35.48±2.97	35.53±3.34	34.67±3.80
			DA	**41.23±2.62** (10 Dim)						
PathMINST (9 classes)	36.22±4.77	37.69±3.53	SVD	28.83±5.01	36.17±4.08	37.72±3.45	37.96±3.72	37.50±3.53	37.76±3.64	37.74±3.59
			NMF	28.67±4.26	36.69±3.04	**39.76±3.10**	39.07±3.33	38.67±2.42	37.54±2.95	37.04±3.29
			DA	**41.43±4.19** (8 Dim)						
TissueMNIST (8 classes)	24.42±3.67	23.65±2.22	SVD	19.02±3.15	20.94±2.74	22.60±2.39	23.21±2.53	23.83±2.44	23.75±2.21	23.75±2.21
			NMF	18.50±3.63	22.40±2.43	**24.58±2.22**	24.23±2.39	23.31±2.58	22.96±2.63	23.02±1.72
			DA	**38.35±5.59** (7 Dim)						

In particular, 5 samples from each class in each dataset are used for training, i.e., forming the "C-way 5-shot" setting (recall C is the number of classes in each dataset). Dim stands for dimensions.

References

1. Donald H. Foley and John W Sammon. An optimal set of discriminant vectors. *IEEE Transactions on computers*, 100(3):281–289, 1975.

2. John W Sammon. An optimal discriminant plane. *IEEE Transactions on Computers*, 100(9):826–829, 1970.

3. Johannes Leuschner, Maximilian Schmidt, Pascal Fernsel, Delf Lachmund, Tobias Boskamp, and Peter Maass. Supervised non-negative matrix factorization methods for maldi imaging applications. *Bioinformatics*, 35(11):1940–1947, 2019.

4. Mukund Balasubramanian and Eric L Schwartz. The isomap algorithm and topological stability. *Science*, 295(5552):7–7, 2002.

5. Andrew Janowczyk and Anant Madabhushi. Deep learning for digital pathology image analysis: A comprehensive tutorial with selected use cases. *Journal of pathology informatics*, 7, 2016.

6. Jun Cheng. brain tumor dataset, Apr 2017.

7. Xuehai He, Xingyi Yang, Shanghang Zhang, Jinyu Zhao, Yichen Zhang, Eric Xing, and Pengtao Xie. Sample-efficient deep learning for covid-19 diagnosis based on ct scans. *medrxiv*, 2020.

8. The 1st diabetic retinopathy – classification of fundus images according to the severity level of diabetic retinopathy.

9. Andrea Acevedo, Anna Merino, Santiago Alférez, Ángel Molina, Laura Boldú, and José Rodellar. A dataset of microscopic peripheral blood cell images for development of automatic recognition systems. *Data in Brief, ISSN: 23523409, Vol. 30,(2020)*, 2020.

10. Walid Al-Dhabyani, Mohammed Gomaa, Hussien Khaled, and Aly Fahmy. Dataset of breast ultrasound images. *Data in brief*, 28:104863, 2020.

11. Philipp Tschandl, Cliff Rosendahl, and Harald Kittler. The ham10000 dataset, a large collection of multi-source dermatoscopic images of common pigmented skin lesions. *Scientific data*, 5(1):1–9, 2018.

12. Daniel S Kermany, Michael Goldbaum, Wenjia Cai, Carolina CS Valentim, Huiying Liang, Sally L Baxter, Alex McKeown, Ge Yang, Xiaokang Wu, Fangbing Yan, et al. Identifying medical diagnoses and treatable diseases by image-based deep learning. *Cell*, 172(5):1122–1131, 2018.

13. Patrick Bilic, Patrick Ferdinand Christ, Eugene Vorontsov, Grzegorz Chlebus, Hao Chen, Qi Dou, Chi Wing Fu, Xiao Han, Pheng-Ann Heng, Jürgen Hesser, et al. The liver tumor segmentation benchmark (LiTS). *arXiv preprint arXiv:1901.04056*, 2019.

14. Jakob Nikolas Kather, Johannes Krisam, Pornpimol Charoentong, Tom Luedde, Esther Herpel, Cleo-Aron Weis, Timo Gaiser, Alexander Marx, Nektarios A Valous, Dyke Ferber, et al. Predicting survival from colorectal cancer histology slides using deep learning: A retrospective multicenter study. *PLoS medicine*, 16(1):e1002730, 2019.

15. Andre Woloshuk, Suraj Khochare, Aljohara F Almulhim, Andrew T McNutt, Dawson Dean, Daria Barwinska, Michael J Ferkowicz, Michael T Eadon, Katherine J Kelly, Kenneth W Dunn, et al. In situ classification of cell types in human kidney tissue using 3d nuclear staining. *Cytometry Part A*, 99(7):707–721, 2021.

16. Jake Snell, Kevin Swersky, and Richard Zemel. Prototypical networks for few-shot learning. *Advances in Neural Information Processing Systems*, 30, 2017.

17. Brenden M Lake, Ruslan Salakhutdinov, and Joshua B Tenenbaum. The omniglot challenge: a 3-year progress report. *Current Opinion in Behavioral Sciences*, 29:97–104, 2019.

18. Diederik P Kingma and Jimmy Ba. Adam: A method for stochastic optimization. *arXiv preprint arXiv:1412.6980*, 2014.
